# Interaction between the number of KIV2 repeats and the cholesterol content of an Lp(a) particle: What to do with these novel data?

**DOI:** 10.1016/j.jlr.2026.101111

**Published:** 2026-07-23

**Keywords:** Lp(a), LCL-C, Lp(a)-C, lipids, lipoproteins, LPA KIV-2

In 1963, the geneticist Kare Berg discovered a new antigen that was associated with low-density lipoproteins (LDL) (1, 2). Berg quickly used family studies to demonstrate that Lp(a) behaved as a genetic trait, and as early as 1974, he and his colleagues reported a higher frequency of elevated Lp(a) levels in patients with coronary heart disease compared with healthy controls (3). Over the next few decades, a small number of investigators focused on the metabolism of Lp(a) in humans, the development of better assays, and the molecular characterization of the *LPA* gene (4). The availability of improved human plasma assays (5) enabled several groups to confirm the association between Lp(a) concentrations and atherosclerotic cardiovascular (ASCVD) in large cohorts, while the cloning of the gene (6) and the identification of the importance of the number of *LPA* KIV2 repeats as the major determinant of Lp(a) levels in the circulation (7) significantly strengthened the association between Lp(a) and risk for ASCVD (8). Progress in all of these areas led to interest by pharmaceutical companies in the development of targeted inhibitors of Lp(a) synthesis and subsequent initiation of large ASCVD outcome trials (9).

Those epidemiological, genetic, and therapeutic advances have been paralleled by studies of the formation of Lp(a), its metabolism in the circulation, and its composition (10, 11). Progress in the latter area was challenging because of the *LPA* KIV-2 repeat-driven heterogeneity, barriers in the methods used to isolate Lp(a) from human plasma, and assay standardization limitations (10). Studies have examined the cholesterol content of the Lp(a) particle [Lp(a)-C], but results were variable (12, 13, 14). In this issue of the *Journal of Lipid Research* “Tsimikas and Marcovina (15) used their recently validated assay for the direct measurement of Lp(a)-C (16) and examined its relationship with apo(a) isoform size. As expected, Lp(a)-C concentration was positively correlated with Lp(a) particle concentration and negatively correlated with apo(a) isoform size, consistent with the well-established inverse relationship between Lp(a) particle number and apo(a) isoform size. Unexpectedly, a different relationship emerged when the authors used their new capture assay to estimate cholesterol content per Lp(a) particle. Across apo(a) sizesisoform sizes, cholesterol content per Lp(a) particle increased with isoform size. These observations reinforced the well-established concept that apo(a) isoform size is a major determinant of circulating Lp(a) levels, while simultaneously unveiling yet another level of isoform-associated Lp(a) heterogeneity: particle cholesterol content.

In writing this commentary, we decided to focus on three aspects of the manuscript. First, we acknowledge the outstanding effort required to validate the current assay, using tools developed decades ago by Marcovina *et al.* (17, 18). The present study determined the effect of the number of apo(a) KIV2 repeats on the cholesterol content of Lp(a) in a cohort of 94 subjects with a wide range of Lp(a) levels and repeats. In presenting their findings, it was unclear why after explaining the calculation of the molar ratio of Lp(a)-C to total Lp(a) using nmol/L for both the numerator [Lp(a)-C] and denominator [Lp(a)], the authors also present the Lp(a)-C/Lp(a)-apoB ratio using mg/dl for both measurements. Although in the United States, mg/dl are the units used for both cholesterol and apoB, the arithmetic manipulation to convert a ratio based on nmol/L to one utilizing mg/dl provides no added information, and the presentation of both ratios in Tables 1 and 3 as well as in the figures might lead a casual reader to conclude that the ratios provide different information. Nevertheless, the findings support a need for laboratories to convert apoB levels to nmol/L (10). The molar ratio is more clinically meaningful because it reflects the cholesterol content per Lp(a) particle, whereas the mg/dl ratio depends on assumptions about the molecular weight of apoB-100. Expressing the Lp(a)-apoB in molar units would also allow clinicians to estimate the proportion of total apoB-containing lipoproteins attributable to Lp(a), an especially useful metric in patients with extremely low LDL-C levels achieved with intensive lipid-lowering therapy, such as statins combined with PCSK9 inhibitors.

Second, the identification of differences in Lp(a)-C content across apo(a) isoform sizes generates several testable hypotheses that can be explored both in vitro and in vivo studies. The authors propose several potential mechanisms to explain the higher Lp(a)-C content observed in particles with larger apo(a) isoforms and the lower Lp(a)-C content in those with smaller isoforms. Additional possibilities merit consideration. For example, it is clear from numerous studies that the liver secretes very low-density lipoproteins (VLDL) with a range of size and triglyceride (TG) content. Thus, smaller apo(a) isoforms might preferentially cross-link to apoB on large, TG-rich VLDL particles within or at the surface of hepatocytes that will become, via lipoprotein lipase (LPL)-mediated lipolysis, apo(a)-containing TG-rich remnant particles in plasma that carry more cholesterol per particle than conventional LDL. Alternatively, larger apo(a) isoforms might preferentially associate with smaller, less TG-rich VLDL particles in the liver that, after secretion, are rapidly converted by LPL to apo(a)-containing LDL-like particles. Overall, the greatest impact of the work by Tsimikas and Marcovina is likely to come from the mechanistic studies that will be stimulated by their findings.

Third, will the demonstration of an association between apo(a) isoform size and the Lp(a)-C per particle alter the way we think about ASCVD risk and plasma Lp(a) levels? Lp(a) particles are structurally defined by the presence of a single apoB100 molecule covalently bound to apo(a) and therefore, represent a subset of apoB-containing lipoprotein particles. The Lp(a) subset differs from the other subsets (VLDL, IDL, and LDL) in that the levels of Lp(a) are tightly linked to the number of genetically determined apo(a) KIV2 repeats, which vary from 1-40, with smaller isoforms associated with greater numbers of Lp(a) particles and larger isoforms with lower numbers of Lp(a) particles. Current research shows that all Lp(a) particles, irrespective of apo(a) isoform, have one molecule of apoB, and numerous studies have shown that the total number of apoB particles in the circulation is the best predictor of ASCVD risk (19, 20, 21). This raises the question: What additional information is provided by knowing the total Lp(a)-C level or, more relevant to the data presented by Tsimikas and Marcovina, by knowing that Lp(a)-C/Lp(a) varies inversely with isoform size? The strong correlation between Lp(a)-C and Lp(a) molar concentrations observed in this study further underscores that Lp(a)-C is primarily a surrogate for particle number. Specifically, the correlations presented in this paper indicate that greater numbers of small apo(a) isoform Lp(a) particles, with less cholesterol per particle, are the main determinants of total Lp(a) or total Lp(a)-C concentrations compared with a smaller number of large apo(a) isoform Lp(a) particles with more cholesterol per particle.

In summary, while this work provides novel biochemical insights into Lp(a) particle heterogeneity, it remains to be formally tested if the measurement of Lp(a)-C offers clinically actionable information beyond established apoB or Lp(a) particle-based metrics. Future studies in large, controlled populations will be required to determine whether determination of Lp(a)-C or the Lp(a)-C/Lp(a) ratio adds value to cardiovascular risk assessment, particularly in the context of the impact of apo(a) isoform size of the cholesterol content per particle.
